# Gastric Electrical Stimulation Has an Effect on Gastric Interstitial cells of Cajal (ICC) That is Associated With Mast Cells

**DOI:** 10.7759/cureus.11458

**Published:** 2020-11-12

**Authors:** Alex Pontikos, Priyanga Jayakumar, Cristian Rios Perez, Heather Barker, Michael Hughes, Xiu Yang, Mostafa Fraig, Abigail Stocker, Lindsay McElmurray, Christina Pinkston, Abell Thomas

**Affiliations:** 1 Gastroenterology, University of Louisville, Louisville, USA; 2 Gastroenterology and Hepatology, University of Louisville, Louisville, USA

**Keywords:** interstitial cells of cajal, mast cells, gastric electrical stimulation, inflammation, gastroparesis

## Abstract

Introduction

Gastric electrical stimulation (GES) is an emerging therapy for gastric motility disorders, showing improvement of gastroparesis related symptoms in previous studies. Interstitial cells of Cajal (ICC) and mast cells have been shown to have a relevant role in gastroparesis pathogenesis. However, the exact effects of GES in those cells is relatively unknown.

Methods

Full thickness biopsies (FTBx) of 20 patients with refractory gastroparesis were obtained at the time of GES placement and repeated when the device was exchanged (mean of 22.5 months between biopsies). A patient-reported outcomes survey was obtained during each office visit during this period. All biopsies were stained with cluster of differentiation 117 (CD117), S100, and mast cell tryptase antibodies and were analyzed.

Results

Half of the patients had a significant increase of ICC during the repeated biopsy compared with baseline (p=0.01) and the other half had significant decrease in ICC levels (p=0.006) but there was no noticeable difference in mast cells counts at baseline between groups. Mast cells analysis was performed in two different groups depending on ICC change from the baseline biopsy (CD117 increase vs CD117 decrease). There was only a significant increase of mast cells count within the CD117 worsened ICC group (p=0.007).

Conclusion

No significant increase in the number of mast cells count seen in patients who received a GES may indicate an improvement in overall inflammation in patients with refractory gastroparesis after GES placement.

## Introduction

Gastric electrical stimulation (GES) is primarily used in patients who have gastric motility disorders characterized by delayed gastric emptying without signs of mechanical obstruction [1]. GES therapy with the Medtronic Enterra system (Medtronic Inc., Minneapolis, MN) has been reported to reduce the clinical symptoms of gastric motility disorders like gastroparesis which is characterized by nausea, vomiting, early satiety, bloating, anorexia, and abdominal pain [2].

The stomach triggers patterns of electromechanical activity through long-lasting waves of depolarization that begin in the corpus and slowly move down to the duodenum, which is termed the slow wave [3]. Gastric slow waves are myogenic in nature and persist even after blocking the nervous activity by isolating the stomach. ICC are electrically paired with smooth muscle cells so that depolarization is linked between ICC and muscle. If the depolarization is large enough to activate the smooth muscle L-type Ca2+ channels, then a contraction occurs [4]. This suggests that ICC has an important role in peristalsis of the stomach and the pathogenesis of gastroparesis. Wang et al. observed a marked reduction in the density of the intramuscular ICC and ICC located at submucosal border of the circular muscle layer at the antrum of rats with induced diabetes mellitus and decreased gastric emptying [5].

The exact mechanism of action of GES and its effect on the interstitial cells of Cajal (ICC) is relatively unknown, but previous studies hypothesized that GES acts via several mechanisms: early and durable gastric prokinetic effect, early anti-inflammatory effect, and early anti-arrhythmic effect [6]. Animal models in diabetic rats showed an increase of c-kit+ cells (molecular marker for ICC) within intramuscular and myenteric layers of antrum after long-pulse GES implant [7]. Mast cells are localized in the gastrointestinal tract and play a key role in the inflammatory process. Their activation could lead to the release of pro-inflammatory mediators without degranulation [8]. A number of studies have documented an increased number of mast cells in the gastrointestinal mucosa in patients with several gastrointestinal diseases, particularly in functional gastrointestinal disorders [9,10].

We hypothesized that permanent GES might have an effect on ICC cells and that actions of GES may correlate to the number of mast cells seen on full thickness biopsies (FTBx) of the stomach after stimulation.

## Materials and methods

The study population was comprised of 20 patients with a majority of females (n=19, 95%) and a mean age of 40.2 years. All patients had FTBx with GES placement for symptoms of gastroparesis uncontrolled with the conventional dietary and medication recommendation (the Initial System) and then subsequently had another FTBx (the Repeat System) when GES was replaced for technical reasons (usually symptoms of electrical shocking). 

**Figure 1:**
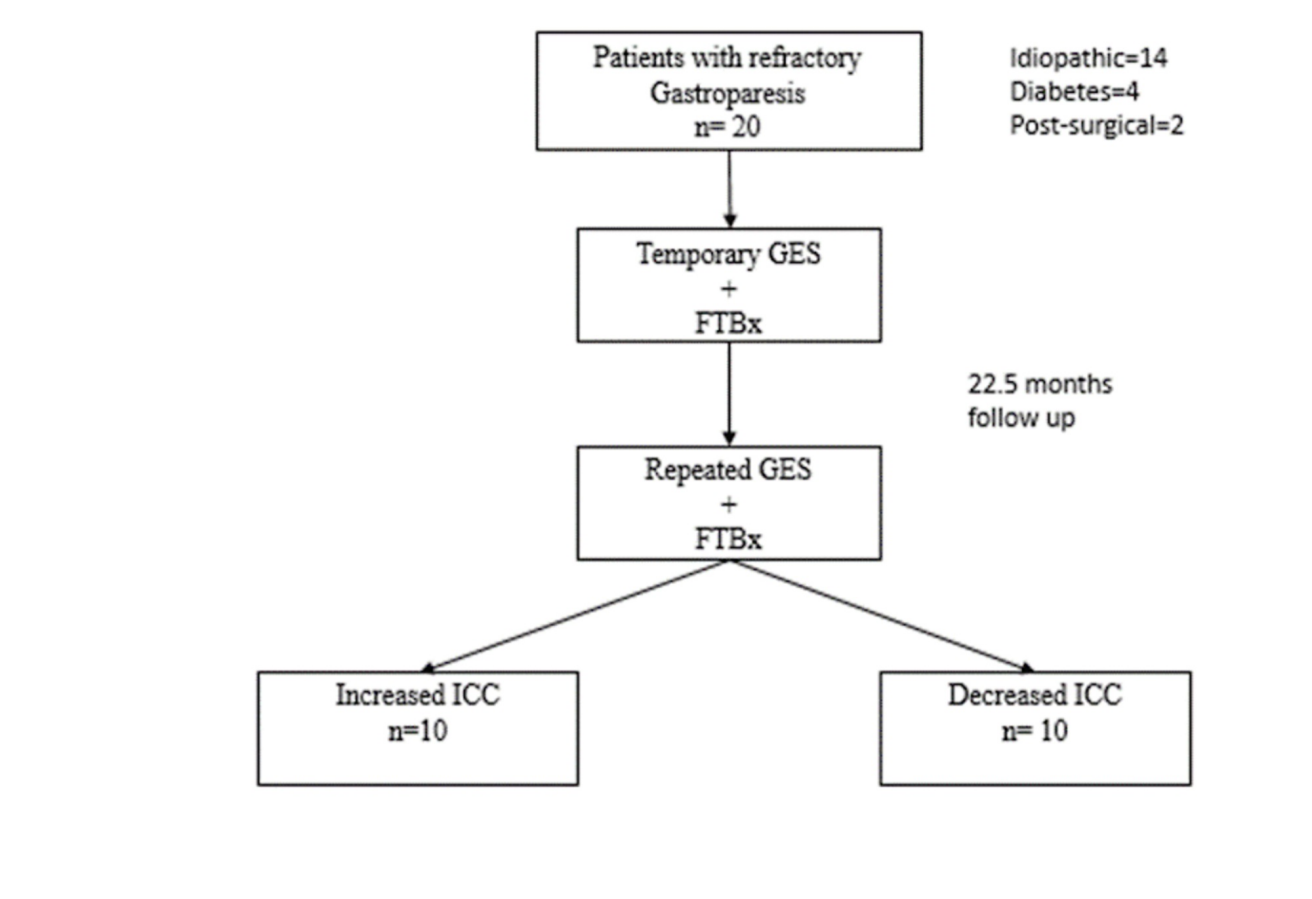
Study population GES: gastric electrical stimulation; FTBx: full thickness biopsy; ICC: interstitial cells of Cajal.

Among the patients, the underlying etiology of gastroparesis was idiopathic in 70% (n=14), diabetes mellitus in 20% (n= 4), and post-surgical in 10% (n=2), which is a similar representation of individuals who received GES for gastroparesis in larger studies. A mean of 22.5 months occurred from the initial system until the repeat system (time between biopsies) (Figure 1).

This study was approved by the University of Louisville Institutional Review Board under studies #17.0662 and #17.0673. All patients signed a standard informed consent prior to placement of the GES devices, both temporary and permanent.

Data collection and analysis

Data was collected prospectively on all the subjects regarding their gastrointestinal symptoms using a standardized traditional patient reported outcomes (PRO) survey. The FTBx specimens were stained with cluster of differentiation 117 (CD117), S100, and mast cell tryptase antibodies. Appropriate positive controls were included for all specific stains. Two pathologists were blinded to clinical history and analyzed the biopsy specimens. The number of ICC cells per high power field (HPF, at 400x magnification) was counted and averaged over 10 fields within the muscularis propria. Gastrointestinal (GI) symptoms, by standardized PRO as above, were measured at baseline and after each GES system implant. Results were compared by paired t-test for FTBx and with a mixed model adjusted for multiple comparisons for GI symptoms and reported as mean and standard error (SE). A p-value of 0.05 was considered statistically significant and all analyses were performed using SAS statistical software, version 9.4 (SAS Institute, Cary, NC, USA).

## Results

**Figure 2:**
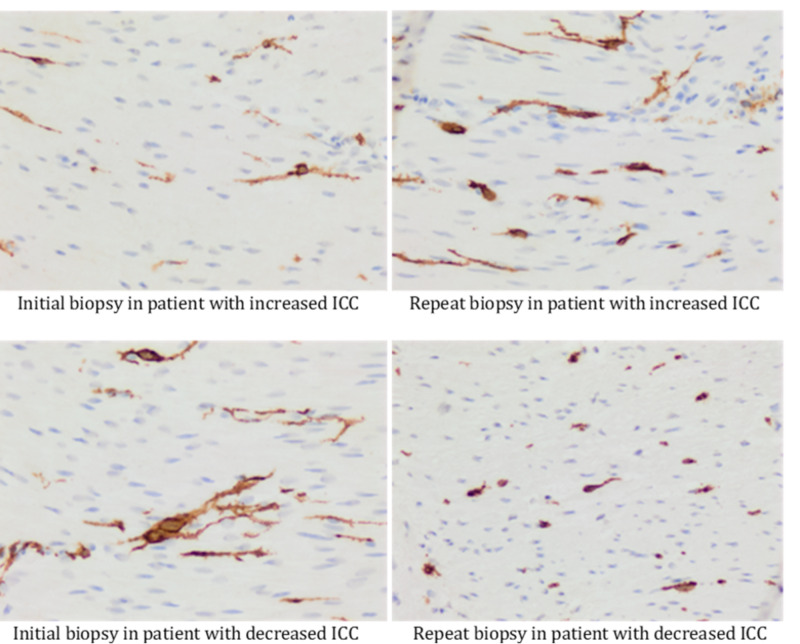
Interstitial cells of Cajal (ICC) counts in full thickness biopsies Full thickness GI biopsies were stratified by whether they increased or decreased ICC counts from baseline. Left upper panel is an initial biopsy and the right upper panel is a repeat biopsy in a patient in the increased ICC group. The left lower panel is an initial biopsy and the right lower panel is a repeat biopsy in a patient in the decreased ICC group.

Patients were stratified based on the change in ICC from baseline to repeat full thickness biopsy. Those with an increase in ICC (n=10) and those with a decrease in ICC (n=10) at the time of repeat full thickness biopsy (See Figure 1-2). Comparisons of CD117 values between initial and repeat biopsies revealed changes in ICC were significantly increased (padj=0.01) or decreased (padj=0.006) in the improving ICC and worsening ICC groups, respectively. Initial CD117 were significantly lower in the improving group at baseline (padj<0.001) but were borderline significantly different with repeated biopsy (padj=0.08). Similar comparisons of the mast cells showed no statistically significant change in counts among the improving group, but showed significant increases in mast cells among those with worsening ICC counts (padj=0.007). Although there were no noticeable differences in mast cells counts at baseline, by the time of the repeated biopsy, the worsening group’s mast cell counts were significantly higher than those of the improving group (padj=0.04).

The group with increased ICC had lower GI symptoms than the decreased ICC group, but there was no statistically significant difference in symptoms between the two groups (all p > 0.05). See data in Table 1 and representative ICCs in Figure 2.

**Table 1:** ICC and mast cell counts for the two patient groups ICC: interstitial cells of Cajal; CD117: cluster of differentiation 117; SE: standard error.

	CD117 Increased ICC		CD117 Worsened ICC		Between Group P-value
	Mean	SE	Mean	SE	
CD117					
Initial biopsy	2.00	0.31	3.90	0.31	<0.001
Repeated biopsy	3.43	0.31	2.34	0.31	0.08
Within Group P-Value	0.01		0.006		
Mast Cells					
Initial biopsy	2.42	1.04	3.29	0.99	0.93
Repeated biopsy	4.27	0.99	8.15	0.99	0.04
Within Group P-Value	0.57		0.007		

## Discussion

The effect of GES on ICC cells and mast cells is not well defined. We found, in this group of 20 patients, that GES was associated with increased ICC counts in about one-half of the patients. A recent study has reported that GES has improved the regeneration of ICCs in diabetic rats, which enhanced delayed gastric emptying [7], but to our knowledge, this is the first study that reports increasing ICC levels after GES placement in patients.

Increased levels of ICC, in our study, could be related to less inflammation, indirectly measured by mast cells levels, and partially attributed to GES. Prior studies reported improvement of serologic markers for inflammation with long-term electrical stimulation on the GI tract of animals and humans [11,12]. A previous study in mice showed delayed gastric emptying was associated with increased production of proinflammatory and reduced production of anti-inflammatory factors by macrophages, leading to loss of ICC [12].

Worsened levels of ICC were related with a significant increase of mast cell count (p=0.007). Also, there was an increase in the number of mast cells during repeated biopsy in both groups, but only was statistically significant in the group with worsened ICC (p=0.004). This data suggest that GES does not control all the inflammation within the population studied. The effect of GES in alternative pathways of inflammation seen in gastroparesis that does not involve mast cells should be studied.

It is crucial to understand the effect of GES on ICC, not only because of their important role in the pathogenesis of gastroparesis, but also because ICC count could predict improvement of gastroparesis symptoms in patients with neurostimulation. Omer et al. observed that patients with normal to moderate depletion of ICC showed better improvement in vomiting and bloating after GES therapy compared with patients who have severe depletion [13].

## Conclusions

Future prospective studies, with larger sample sizes, and additional measures of inflammation are warranted to explore the observation that electrical stimulation may be associated with increased ICC counts in some patients.

## References

[1] McCallum RW, Lin Z, Forster J, Roeser K, Hou Q, Sarosiek I (2011). Gastric electrical stimulation improves outcomes of patients with gastroparesis for up to 10 years. Clin Gastroenterol Hepatol.

[2] Yin J, Abell TL, McCallum RW, Chen JDZ (2012). Gastric neuromodulation with Enterra system for nausea and vomiting in patients with gastroparesis. Neuromodulation.

[3] Hirst GDS, Edwards FR (2006). Electrical events underlying organized myogenic contractions of the guinea pig stomach. J Physiol.

[4] Farrugia G (1999). Ionic conductances in gastrointestinal smooth muscles and interstitial cells of Cajal. Annu Rev Physiol.

[5] Wang XY, Huizinga JD, Diamond J, Liu LWC (2009). Loss of intramuscular and submuscular interstitial cells of Cajal and associated enteric nerves is related to decreased gastric emptying in streptozotocin-induced diabetes. Neurogastroenterol Motil.

[6] Abell TL, Kedar A, Stocker A (2019). Gastroparesis syndromes: response to electrical stimulation. J Neurogastroenterol Motil.

[7] Chen Y, Wang H, Li H, Liu S (2018). Long-pulse gastric electrical stimulation repairs interstitial cells of Cajal and smooth muscle cells in the gastric antrum of diabetic rats. Gastroenterol Res Pract.

[8] Theoharides TC, Alysandratos KD, Angelidou A (2012). Mast cells and inflammation. Biochim Biophys Acta.

[9] Barbara G, Stanghellini V, De Giorgio R, Corinaldesi R (2006). Functional gastrointestinal disorders and mast cells: implications for therapy. Neurogastroenterol Motil.

[10] Ramsay DB, Stephen S, Borum M, Voltaggio L, Doman DB (2010). Mast cells in gastrointestinal disease. Gastroenterol Hepatol (N Y).

[11] Li H, Chen Y, Liu S, Hou XH (2016). Long-pulse gastric electrical stimulation protects interstitial cells of Cajal in diabetic rats via IGF-1 signaling pathway. World J Gastroenterol.

[12] Cipriani G, Gibbons SJ, Miller KE (2018). Change in populations of macrophages promotes development of delayed gastric emptying in mice. Gastroenterology.

[13] Omer E, Kedar A, Narajarao H (2019). Cajal cell counts are important predictors of outcomes in drug refractory gastroparesis patients with neurostimulation. J Clin Gastroenterol.

